# Efficacy and safety of saroglitazar in real‐world patients of non‐alcoholic fatty liver disease with or without diabetes including compensated cirrhosis: A tertiary care center experience

**DOI:** 10.1002/jgh3.12878

**Published:** 2023-02-20

**Authors:** Sujit Chaudhuri, Agnibha Dutta, Sunil Baran Das Chakraborty

**Affiliations:** ^1^ Department of Gastroenterology AMRI Hospitals Kolkata India

**Keywords:** cirrhosis, fibrosis, NAFLD, saroglitazar

## Abstract

**Background and Aim:**

Saroglitazar, a dual PPAR α/γ agonist, is useful in management of NAFLD and diabetic dyslipidemia. Here, we report the safety and efficacy of saroglitazar in NAFLD patients with or without diabetes including compensated cirrhosis.

**Methods:**

Patients, started on saroglitazar 4 mg were prospectively evaluated for 52 weeks in a tertiary care center in Eastern India. Effectiveness was measured in terms of anthropometric measurements, fasting blood glucose, LFT, lipid profile, HbA1c, and elastography parameters (LSM and CAP) measured at baseline, 24, and 52 weeks. Adverse drug reactions were monitored.

**Results:**

A total of 112 patients were enrolled in the study, of whom 63 patients were taken up for per‐protocol analysis. Mean age was 49.11 ± 11.09 years and 46(73%) were male. Thirty‐four (54%) were nondiabetic. Eleven patients had compensated cirrhosis. There was significant improvement of LSM from baseline (11.03 ± 7.19 kPa) to 24‐week (9.29 ± 6.39 kPa) and 52‐week (8.59 ± 6.35 kPa) values respectively (*P* < 0.001). Significant reduction was also seen in CAP values, ALT, AST, HbA1c, LDL, total cholesterol, and triglyceride values. There was no significant weight change along the study interval. Pruritus occurred in one patient who required treatment discontinuation and another patient had mild symptomatic loose motion.

**Conclusions:**

Saroglitazar is effective and safe in improving biochemical parameters and LSM and CAP values irrespective of weight reduction. It may be used in compensated cirrhotics with close monitoring for side effects.

## Graphical abstract

One hundred and twelve NAFLD patients were screened and 76 patients, of whom 11 had compensated cirrhosis (LSM ≥ 14 kPa), were started on saroglitazar 4 mg. There was significant improvement of LSM from baseline (11.03 ± 7.19 kPa) to 24‐week (9.29 ± 6.39 kPa) and 52‐week (8.59 ± 6.35 kPa) values respectively. The favorable response was seen across all the fibrosis stages as categorized based on LSM values (F0–F1: LSM <7 kPa; F2: LSM 7–10 kPa; F3: LSM ≥10 to <14 KPa and F4: LSM ≥14 kPa). Significant reduction was also seen in CAP, ALT, AST, HbA1c, LDL, total cholesterol, and triglyceride values. There was no significant weight change along the study interval. Pruritus occurred in one patient who required treatment discontinuation.

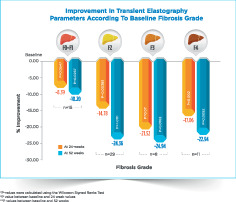

## Background and aim

Non‐alcoholic fatty liver disease (NAFLD) is a modern‐day epidemic. Its prevalence is almost 30% in Asian countries.^1^ It is a spectrum of illness from non‐alcoholic fatty liver to steatohepatitis (NASH) with a chance of progression to fibrosis ultimately leading to cirrhosis. This entity is being increasingly a cause of cirrhosis and hepatocellular carcinoma (HCC) globally.

Pathogenesis of NAFLD is multifactorial. NASH often develops in the context of excess adiposity and systemic insulin resistance.^2^ Pathogenesis involves simple accumulation of fat followed by a variable contribution from different pathogenic drivers, such as lipotoxicity, oxidative stress, inflammation, and immune system activation. Insulin resistance leads to lipid accumulation in hepatocyte as the first hit and is followed by a second hit driven by lipotoxic metabolite‐induced mitochondrial dysfunction and oxidative stress leading to hepatocyte death and inflammation.^3^

Lifestyle modification with exercise and achieving weight loss remains the cornerstone of therapy for NAFLD, but it is very difficult to sustain for a prolonged period. Despite better understanding of the pathogenesis and progression of NAFLD‐/NASH‐related liver disease, drug targeting key areas of metabolic pathways is lacking. In the last decade or so, multiple drugs have been tested for this condition, but till now a reliable medicine with a definite therapeutic end point is lacking. Also, in advanced stages of the disease, lifestyle modification alone is unlikely to be adequate management and may not be justifiable as monotherapy.

Saroglitazar is a dual potent PPAR‐α/γ agonist. Synergistic effect of improved lipid oxidation and improved insulin resistance by PPAR‐α and PPAR‐γ, respectively, makes rational its use in NASH/NAFLD. Its efficacy in management of patients of NAFLD and diabetic dyslipidemia is being reported throughout the world.^4^, ^5^, ^6^, ^7^ Furthermore, data regarding efficacy and safety of saroglitazar in non‐diabetics and in patients with compensated cirrhosis are lacking.

Diagnosis of baseline severity and assessment of therapeutic response of NAFLD have predominantly shifted to noninvasive methods. Liver biopsy, although considered gold standard, has its drawbacks, such as being invasive and less patient acceptance, particularly in repeated testing.

We aimed to evaluate the real‐world safety and efficacy of saroglitazar 4 mg in patients with NAFLD primarily by seeing the improvement in liver steatosis and stiffness.

## Method

This is an investigator‐initiated, single‐center, prospective, observational, open‐label, single‐arm study to evaluate the safety and efficacy of saroglitazar 4 mg in patients with NAFLD/NASH in real‐life setting, conducted in a tertiary care research institute in Kolkata.

Ethical clearance was obtained from institutional ethical committee.

### Inclusion criteria

The included patients were of age ≥18 years, and diagnosed for NAFLD fulfilling the AASLD guidelines,^8^ hepatic steatosis by imaging or histology, absence of significant alcohol consumption, competing etiologies for hepatic steatosis, and co‐existing causes for chronic liver disease, having elevated ALT levels along with liver stiffness value ≥6 kPa and/or liver steatosis CAP >290 dB/m, measured through FibroScan/TE, has been included in the study.

### Exclusion criteria

The patients with any evidence of alcoholic liver disease, significant alcohol use (210 gm/week in male, 140 g/week in females), concomitant use of any steatogenic drugs, and clinical or lab evidence of other liver disease have also been excluded from the study. Patients with decompensated cirrhosis have been excluded. Other liver illnesses like chronic hepatitis B or C infection, Wilson's disease, and drugs causing liver fibrosis like amiodarone, methotrexate, etc., have been excluded. Patients taking thiazolidine diones or saroglitazar in the last 6 months were also excluded.

The patients found to have any confounding factors, which may overestimate the FibroScan values like liver congestion, ascites, liver inflammation due to any recent liver illness or drinking alcohol, benign or cancerous tumors in the liver, biliary obstructions, etc., were also excluded from the study.

The person who has known allergy, sensitivity or intolerance to saroglitazar or formulation ingredients, women with pregnancy or lactation or of childbearing potential and not using appropriate contraceptive measures, has history or other evidence of severe illness or any other conditions that would make the patient, in the opinion of the investigator, unsuitable for the study and those who used vitamin E ≥ 800 IU/day or multivitamins containing vitamin E ≥ 800 IU/day in the 1 month preceding screening visit, were also excluded from the study.

Saroglitazar 4 mg once daily was prescribed in routine clinical practice to eligible patients, who visited the outpatient department of AMRI Hospital, at Salt Lake, Kolkata in April 2019, and fulfilled the inclusion/exclusion criteria, along with routine care of diet and lifestyle modification. Informed consent was taken from every participant. The patients were followed up at an interval of 3 months and up to a total of 1‐year duration and the safety and efficacy data were collected in an excel sheet at every follow‐up visit. The data were collected by interviewing every participant using a set of questionnaires related to demographic features like age, gender, anthropometric measurement, family history for liver disease or other metabolic disorder, ongoing medications, detailed medical history, including personal history of alcohol intake, and any comorbidities like dyslipidemia, diabetes, and hypertension. A trained technical assistant did all the physical examination. Height was measured by a standard stadiometer, and weight was measured using a standard bathroom scale. Anthropometric measurements were cross‐checked to ensure inter‐observer reliability. Body mass index (BMI) was calculated using height in meter and weight in kg.

Baseline patients demographics like age, gender, weight, height, waist circumference, co‐morbid conditions, and ongoing medications were recorded.

Detailed medical history, including personal history of alcohol intake, concomitant medication, and presence of metabolic comorbidities like obesity, hypertension, diabetes mellitus, coronary artery, and cerebrovascular disease, has been recorded. Family history of any metabolic or liver‐related disorders has also been captured in the excel sheet at the baseline visit.

The EchosensFibroScan® 530 Compact machine has been used to measure both fibrosis (liver stiffness) and steatosis (ultrasound attenuation rate/ CAP), together by an expert and Echosens certified technician. M and XL probes were used for subjects with BMI less than 30 kg/m^2^ and ≥ 30 kg/m^2^ respectively.

At baseline, patients were categorized into four categories based on liver stiffness (LSM) value: (i) F0–F1: LSM <7 kPa; (ii) F2: LSM 7–10 kPa; (iii) F3: LSM >10 to <14 KPa; and (iv) F4: LSM >14 kPa.

Patients in the last group also underwent upper GI endoscopy for variceal screening.

The patients on continued saroglitazar 4 mg once daily therapy have undergone follow‐up elastography at 24 and 52 weeks. Anthropometric measurements were taken at each clinical visit. Patients underwent testing for lipid profile, LFT, HbA1c, and CBC at baseline, 24 weeks, and 52 weeks. The data were analyzed using paired t‐test on SPSS Version 22 statistical package.

At every follow‐up visit, safety parameters were assessed in the form of history regarding possible side effects as well as clinical examination.

### Statistical analysis

Continuous data with normal distribution were presented as mean and SD and without normal distribution as median and IQR. Normally distributed continuous data were analyzed using paired t‐test. Wilcoxon signed‐rank test was used for all skewed non‐normal data. All categorical data were presented as proportion and analyzed using the χ2 test. *P* values less than 0.05 were considered significant. All the statistical analysis was done on SPSS ver 22 statistical package.

## Results

Between April and October 2019, 112 patients were screened for the study. Thirty‐six had one or more exclusion criteria, treatment had to be stopped in one due to allergic complication, 12 patients did not complete 12‐month therapy and were lost to follow‐up. Sixty‐three patients were taken up for per‐protocol analysis.

Basic demographic profile of patients is shown in Table 1. The mean age of the population was 49.11 ± 11.09 years and 46(73%) were male. Thirty‐four (54%) were nondiabetic.

**Table 1:** Baseline characteristics of the subjects*

Age (Years)^†^	49.11 (11.09)
Weight (Kg)^†^	73.56 (12.47)
BMI (Kg/m^2^)^†^	27.25(4.10)
Waist circumference (meter) mean (SD)	1.02(0.15)
Male, *n* (%)	46 (73.02)
Nondiabetic, *n* (%)	34 (53.97)
Non‐dyslipidemic, *n* (%)	46 (73.02)
Non‐hypertensive, *n* (%)	34 (53.97)
Family history of liver disease	21 (33.33)
LSM (KPa)^‡^	8.5 (3.9)
CAP (dB/m)^‡^	328 (46)
FBS (mg/dl)^‡^	109 (31.5)
HbA1c (%)^‡^	6.45 (1.5)
ALT (IU/L)^‡^	45 (34.5)
AST (IU/L)^‡^	39 (23)
LDL (mg/dl)^‡^	125 (38.4)
TG (mg/dl)^‡^	160 (43.4)

* Categorical variables are expressed as number (%); continuous parametric variables are expressed as mean (SD); and nonparametric (Skewed) variables are expressed as median (IQR).

^†^ Mean (SD).

^‡^ Median (IQR).

ALT, alanine transaminase; AST, aspartate aminotransferase; BMI, body mass index; CAP, controlled attenuation parameter; FBS, fasting blood sugar; LDL, low‐density lipoprotein; LSM, liver stiffness measurement; SD, standard deviation; TG, triglyceride.

Baseline investigations are shown in Table 1. Median ALT and AST values were 45 (18–188) and 39 (25–170) respectively. ALT and AST values were greater than 40 IU/L in 86% (*n* = 55) and 81% (*n* = 52) respectively. Median LSM at baseline was 8.5 (4.2–35.6) kPa. Eleven patients had LSM >14 kPa, of them three were found to have esophageal varices. The median CAP value was 328 (233–400) dB/m.

### Post‐treatment investigation

Compared with baseline, after 24 and 52 weeks of saroglitazar therapy, there was reduction in ALT levels by 37% and 52% respectively, which was statistically significant. Similar trends were seen in AST also. There was statistically significant reduction in cholesterol and triglyceride levels at 52 by 24.1% and 40.6% (Table 2).

**Table 2:** Improvements in secondary end points at weeks 24 and 52

Secondary parameters	Baseline median (IQR)	% Change* (at 24 weeks)	* *P* value (z score)	% Change^#^ (at 52 weeks)	† *P* value (z score)
Weight (kg)	72 (16)	1.05	0.139 (−1.479)	1.42%	0.055 (−1.922)
WC (cm)	101 (14.5)	−2.25	0.038 (−2.075)	−2.97%	0.049 (−1.971)
BMI (kg/m^2^)	26.3 (4.6)	1.03	0.163 (−1.393)	1.31%	0.13 (−1.515)
HDL‐C (mg/dl)	39 (6)	8.42	<0.001 (−4.295)	15.59%	<0.001 (−5.124)
LDL‐C (mg/dl)	125 (38.4)	−15.9	<0.001 (−5.388)	−25.54%	<0.001 (−6.069)
Tot Chol (mg/dl)	178 (47)	−16.5	<0.001 (−5.895)	−24.05%	<0.001 (−6.258)
TG (mg/dl)	160 (43.4)	−29.6	<0.001 (−6.401)	−40.61%	<0.001 (−6.644)
AST (IU/L)	39 (23)	−25.1	<0.001 (−3.982)	−40.78%	<0.001 (−5.752)
ALT (IU/L)	45 (34.5)	−36.7	<0.001 (−5.487)	−52.21%	<0.001 (−6.542)

* % Change in mean values.

^†^ *P* value between baseline and 52‐week data, calculated using Wilcoxon signed‐rank test.

**Figure 1:**
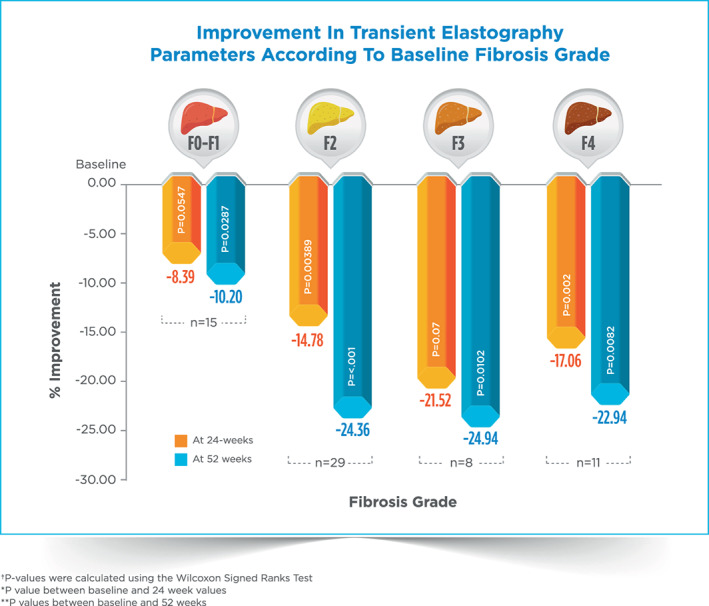
Improvement in liver stiffness measurement at various fibrosis grade after 24 and 52 weeks.

Eleven patients had LSM ≥14 kPa corresponding with F4 fibrosis.^9^ There was statistically significant improvement in LSM value in all the four groups at 52 weeks with decrease of around 22–25% in patients with baseline LSM ≥7 kPa (Table 3 and Fig. 1). There was 14% reduction in CAP values from baseline across all the patient population, which was also statistically significant.

**Table 3:** 24‐ and 52‐week assessment of improvement in transient elastography parameters according to baseline fibrosis grade

Fibrosis grades (LSM) (Number)	Baseline	At 24 week				At 52 weeks			
	Mean ± SD	Mean ± SD	Absolute difference	% Difference	* *p*	Mean ± SD	Absolute difference	% Difference	** *p*
F0–F1 (<7 kPa) (*n* = 15)	6.04 ± 0.73	5.53 ± 1.27	−0.51	−8.39%	0.0547	5.39 ± 1.3	−0.65	−10.82%	0.0287
F2 (≥7 to 10 kPa) (*n* = 29)	8.21 ± 0.82	7.00 ± 2.37	−1.21	−14.78%	0.00389	6.21 ± 1.27	−2.00	−24.36%	<0.001
F3 (≥10 to <14 kPa) (*n* = 8)	11.33 ± 0.76	8.89 ± 3.98	−2.44	−21.52%	0.07	8.5 ± 2.20	−2.83	‐ 24.94%	0.0102
F4 (≥14 kPa) (*n* = 11)	25.05 ± 6.38	20.77 ± 6.54	−4.27	−17.06%	0.002	19.30 ± 9.06	−5.75	−22.94%	0.0082

* *P* value between baseline and 24‐week values.

** *P* values between baseline and 52 weeks.

There was a significant improvement in median CAP at 24 weeks [281 (70) dB/m, *P* < 0.001] and 52 weeks [287 (67) dB/m, *P* < 0.001] as compared with the baseline [328 (46) dB/m] (Table 4).

**Table 4:** Improvement in primary end point (liver stiffness measurement and CAP score) at Weeks 24 and 52

Parameters	At baseline	24 weeks	52 weeks	% Change^‡^	* *P* value (z score)
LSM (kPa)^†^	8.5 (3.9)	6.5 (2.8)	6.5 (2.9)	−22.11%	<0.001 (−5.467)
CAP (dB/m)^†^	328 (46)	281 (70)	287 (67)	−14.18%	<0.001 (−6.278)

* *P* value between baseline and 52‐week data, calculated using Wilcoxon signed‐rank test.

^†^ Median (IQR).

^‡^ % Change in mean values at 52 weeks compared with baseline.

The median LSM at week 24 [6.5 (2.8) kPa, *P* < 0.001] and at week 52 [6.5 (2.9) kPa, *P* < 0.001] was also statistically significantly improved than the baseline median LSM of 8.5 (3.9) kPa (Table 4).

There was no significant weight reduction in the patient population, and BMI distribution was similar at baseline and 52 weeks (Table 5); rather overall there was 1.42% weight gain. However, reduction in HbA1c, LDL and increase in HDL was seen after 52 weeks of therapy (Table 2).

**Table 5:** BMI distribution in subjects at baseline and 52 weeks

Obesity categorization^†^ (BMI range in Kg/m^2^)	At baseline, *n* (%)	After 52 weeks, *n* (%)
Underweight	0 (0)	0 (0)
Normal weight	18 (28.6)	19 (30.2)
Overweight	30 (47.6)	29 (46.03)
Obese (Class I)	8 (12.7)	8 (12.7)
Obese (Class II)	4 (6.3)	4 (6.3)
Obese (Class III)	1 (1.6)	1 (1.6)

^†^ Obesity categorization based on WHO 2000 definition [underweight (<18.5 Kg/m^2^), normal weight (18.5–24.9 Kg/m^2^), overweight (25.0–29.9 Kg/m^2^), Obese Class I (30.0–34.9 Kg/m^2^), Obese Class II (35.0–39.9 Kg/m^2^), Obese Class III (≥40.0 Kg/m^2^)].

Saroglitazar 4 mg once daily was found to be safe and well tolerated, as there was no severe reported drug‐related major adverse event, which results in discontinuation of the therapy, except one case of minor itching, which subsided within 2 weeks of treatment discontinuation and one other case who has reported mild symptomatic loose motion.

## Discussion

This study aimed to test the efficacy and tolerability of saroglitazar in an extended population including nondiabetics and ones having significant fibrosis on elastography. It is shown that saroglitazar shows significant reduction in LSM and CAP values as well as improvement in metabolic parameters. One novel finding is that saroglitazar is tolerable, safe, and efficacious in compensated F4 fibrosis also. There has been limited data regarding the efficacy of saroglitazar in the Indian population. Padole et al.^10^ have shown that saroglitazar is effective in reducing transaminase value, but LSM and CAP reduction was seen only in patients who achieved at least 5% weight reduction. This study refutes the same showing improvement in LSM irrespective of weight change. Further studies with higher sample size are required to throw further light into it.

This study adds to real‐world experience of saroglitazar in the NASH population. It is predominantly recommended for patients with diabetes, but Sarin et al. conducted a study including nondiabetics as well.^11^ This study also included nondiabetic patients and its efficacy is shown in this population as well. There was a significant improvement in hepatic steatosis as measured by improvement in CAP. MR‐PDDF is known to be superior to CAP assessment, but the use of the former is limited by its cost and availability and for real‐world scenario, CAP is a validated parameter for assessment of steatosis. For the assessment of fibrosis and inflammation, biopsy remains the gold standard. But apart from research setting, biopsy for all patients of NASH remains impractical considering the risk–benefit ratio and patient acceptability. Further limitation of biopsy remains sampling bias. Accuracy of LSM to assess fibrosis is an area of ongoing research and there are different studies supporting the same.^12^, ^13^, ^14^, ^15^ For real‐life patients, it remains a feasible and repeatable option. Therefore, it has been used in this study. This study showed improvement in LSM and CAP values mostly in the first six months that persisted after 52 weeks of therapy.

As α effect of saroglitazar is more than γ agonism, lipid profile improvement is expected to be more than the improvement of glycemic status. It was corroborated in the result as well with patients having around 25% improvement in total cholesterol and LDL level and 40% reduction in triglyceride level. As expected from γ agonism, there was weight gain in the patient population despite improvement in LSM and CAP, which is shown in other studies^16^ as well. This area needs further assessment. Weight loss with lifestyle management has been shown to improve NASH. So, the study of effect of medication adjusted for weight gain or loss would require different study design. This may fill the knowledge gap in this domain. Other γ agonism effect in the form of improvement of blood glucose and HbA1c has been shown in the patient population.

Liver enzymes have been used traditionally to assess the extent of liver injury. It has inherent limitations being nonspecific and not correlative with fibrosis. However, it is very useful in practical setting due to its availability and low cost. Study population showed a significant reduction of ALT and AST both at 24 and 52 weeks. It corroborates with recent real‐life data^17^ on saroglitazar in NASH population.

Perhaps this study was the first of its kind to test the efficacy and safety of saroglitazar in compensated cirrhotics. Cirrhosis was diagnosed based on either LSM values or presence of varices on upper GI endoscopy with other causes being excluded. Saroglitazar was well tolerated with no new onset decompensation in this group as well as efficacious with approximately 23% reduction in LSM values at 52 weeks as compared with baseline. This is definitely a novel finding. Although only 11 patients were cirrhotics, further studies with larger patient population are warranted to possibly expand the use of this molecule.

The first strength of the study is its patient population. Inclusion of nondiabetics and cirrhotics allowed to assess efficacy and safety of saroglitazar in this less studied population. The second strength is longer follow‐up duration of 52 weeks where most studies have shorter follow‐up data. The third strength is the use of noninvasive markers at frequent time interval that mimics real‐life experience, which most of the physicians follow.

Limitation of this study remains that it cannot predict superiority over other molecules presently being researched for NASH. Absence of biopsy data would indicate that idea of baseline necro‐inflammatory activity was unknown. Third, follow‐up data were not there on whether discontinuation of drug would lead to reversal of biochemical and stiffness parameter improvement or persistence of benefit of drug. This very important question was not addressed in the study design. Fourth, cardiovascular outcome was not assessed. The cause of mortality and morbidity in NASH population is predominantly cardiovascular. So, whether improvement of metabolic parameter ultimately translates to improvement in cardiovascular outcome or not is a question that would require long‐term follow‐up, for which this study was not designed. Fifth, there was no placebo arm to compare the contribution of lifestyle modification alone to addition of saroglitazar.

## Conclusion

Saroglitazar is effective and safe in improving biochemical parameters and LSM and CAP values irrespective of weight reduction. It may be used in compensated cirrhotics with close monitoring for side effects.

## Ethics statement

The study design was approved by institutional ethical committee of AMRI Hospitals, Salt Lake.

## Patient consent statement

Written consent was obtained from each participant after explaining in their own vernacular, consent forms preserved by the author.

## Data Availability

The patient related data are accessible from principal author on request.

## References

[1] De Roza MA , Goh GB . The increasing clinical burden of NAFLD in Asia. Lancet Gastroenterol. Hepatol. 2019; 4: 333–4. 30902671 10.1016/S2468-1253(19)30093-7

[2] Dowman JK , Tomlinson JW , Newsome PN . Pathogenesis of non‐alcoholic fatty liver disease. QJM. 2010; 103: 71–83. 19914930 10.1093/qjmed/hcp158 PMC2810391

[3] Parthasarathy G , Revelo X , Malhi H . Pathogenesis of Nonalcoholic Steatohepatitis: An Overview. Hepatol Commun. 2020; 4: 478–92. 32258944 10.1002/hep4.1479 PMC7109346

[4] Joshi SR . Saroglitazar for the treatment of dyslipidemia in diabetic patients. Expert Opin Pharmacother. 2015; 16: 597–606. 25674933 10.1517/14656566.2015.1009894

[5] Kaul U , Parmar D , Manjunath K *et al*. New dual peroxisome proliferator activated receptor agonist‐Saroglitazar in diabetic dyslipidemia and non‐alcoholic fatty liver disease: integrated analysis of the real world evidence. Cardiovasc. Diabetol. 2019; 18: 80. 31208414 10.1186/s12933-019-0884-3 PMC6580520

[6] Sosale A , Saboo B , Sosale B . Saroglitazar for the treatment of hypertrig‐lyceridemia in patients with type 2 diabetes: current evidence. Diabetes Metab. Syndr. Obes. 2015; 15: 189–96. 10.2147/DMSO.S49592 PMC4403747 25926748

[7] Krishnappa M , Patil K , Parmar K *et al*. Effect of saroglitazar 2 mg and 4 mg on glycemic control, lipid profile and cardiovascular disease risk in patients with type 2 diabetes mellitus: a 56‐week, randomized, double blind, phase 3 study (PRESS XII study). Cardiovasc. Diabetol. 2020; 19: 93. 32560724 10.1186/s12933-020-01073-w PMC7305598

[8] Chalasani N , Younossi Z , Lavine JE *et al*. The diagnosis and management of nonalcoholic fatty liver disease: Practice guidance from the American Association for the Study of Liver Diseases. Hepatology. 2018; 67: 328–57. 28714183 10.1002/hep.29367

[9] Wong GL , Wong VW , Choi PC *et al*. Assessment of fibrosis by transient elastography compared with liver biopsy and morphometry in chronic liver diseases. Clin. Gastroenterol. Hepatol. 2008; 6: 1027–35. 18456573 10.1016/j.cgh.2008.02.038

[10] Padole P , Arora A , Sharma P , Chand P , Verma N , Kumar A . Saroglitazar for Nonalcoholic Fatty Liver Disease: A Single Centre Experience in 91 Patients. J. Clin. Exp. Hepatol. 2022; 12: 435–39. 35535066 10.1016/j.jceh.2021.06.015 PMC9077151

[11] Sarin SK *et al*. A prospective, multi‐centre, double‐blind, randomized trial of Saroglitazar 4 mg compared to placebo in patients with nonalcoholic steatohepatitis. Hepatol. Int. 2020; 14: S326.

[12] Ducancelle A , Leroy V , Vergniol J *et al*. A Single Test Combining Blood Markers and Elastography is More Accurate Than Other Fibrosis Tests in the Main Causes of Chronic Liver Diseases. J. Clin. Gastroenterol. 2017; 51: 639–49. 28692443 10.1097/MCG.0000000000000788

[13] Das K , Sarkar R , Ahmed SM *et al*. "Normal" liver stiffness measure (LSM) values are higher in both lean and obese individuals: a population‐based study from a developing country. Hepatology. 2012; 55: 584–93. 21952989 10.1002/hep.24694

[14] Yoshioka K , Hashimoto S , Kawabe N . Measurement of liver stiffness as a non‐invasive method for diagnosis of non‐alcoholic fatty liver disease. Hepatol. Res. 2015; 45: 142–51. 25040931 10.1111/hepr.12388

[15] Petta S , Vanni E , Bugianesi E *et al*. The combination of liver stiffness measurement and NAFLD fibrosis score improves the noninvasive diagnostic accuracy for severe liver fibrosis in patients with nonalcoholic fatty liver disease. Liver Int. 2015; 35: 1566–73. 24798049 10.1111/liv.12584

[16] Gawrieh S , Noureddin M , Loo N *et al*. Saroglitazar, a PPAR‐α/γ Agonist, for Treatment of NAFLD: A Randomized Controlled Double‐Blind Phase 2 Trial. Hepatology. 2021; 74: 1809–24. 33811367 10.1002/hep.31843

[17] Goyal O , Nohria S , Goyal P *et al*. Saroglitazar in patients with non‐alcoholic fatty liver disease and diabetic dyslipidemia: a prospective, observational, real world study. Sci. Rep. 2020; 10: 21117. 33273703 10.1038/s41598-020-78342-x PMC7713236

